# Supplementary material for: TBP-Related Factor 2 as a Trigger for Robertsonian Translocations and Speciation
Source: Int J Mol Sci. 2020 Nov 23;21(22):8871. doi: 10.3390/ijms21228871 (PMC7700478; doi:10.3390/ijms21228871)
Supplement: Supplementary file 1 [file ijms-21-08871-s001.zip › Figure_S1.pdf]

cagtcaccacgatgccatcacggatctgctgatgtgcaaggacaagggccagatctacatagcatctgcatcgcgaaa

cggtgtcatcaagctgtggaagtgagcgcccactaaatagtatttaattattcaactgtgattagcctaataaactgcaata

Promoter predicted using BDGP

tgtgtaactatttaataaacggttgcttctaaactgtactctcttgttttaaaaacttggagcttggcgcttcaagcaaaaaaa

Promoter predicted using BDGP

Predicted TSS (matches with known one)

aaaatttgaacacaacataccatattttaaaatagtaggccagccgtctcatgtaGTCACTTCAAACTTGT

Drosophila transcriptional initiator

Bridge

Bridge

D1-RC 1st exon

TTAACTTTTGGACATATGGATAGCTTAACTTGGTTGTTCACGCAAGAAG

GAAGTTCAAAAAATAATTTTTGGTTATTGCTTTAGTTCAAGTTTTTTATA

BRE

TATA-box

AATTTGTTTCTTTTGATAttcctaaactgtaagtagacaatttttacaccttacaatttccaaaagtaaaaa

Drosophila initiator

Bridge

Bridge

gagtttttcacaaaagctacgtttcattttatgttaatacatagttaaaatgcaatatgaccaaatgaaagggaattgtttta c

Promoter predicted using BDGP

Predicted TSS

gcaaacttaaattaaacggcctcaattttgaacgcatcgAtgcagcgcttaatcgatatcaaatcgaaaaaaatatcgc

Drosophila initiator

Bridge

Bridge

TSS+1

D1-RA, D1-RD 1st exon

aaaaattatcgctgctcgatgcaccgacatatttttTTTACATCACTACTTTGAACTGTACTAG

CGTGCGCGTATTTTACAATTACAAATTGAGAAGGCAAAagtaagtaacgcaagta

attccctttcggatctggaaaaactagaatttttaagaattttttgagaatattttttgcagcatttatgcgcattcaagcccta

atgcaaaattgtttttctgtatatttttccag

Drosophila initiator

Bridge

Bridge

Figure1 S1. Sequence analysis of *D1* 5’UTRs.

With green font and dashed lines denoted promoter region predicted using BDGP neural network promoter prediction software. With gray colour denoted exons of D1. TSS – transcriptional start site.
