# Supplementary material for: TBP-Related Factor 2 as a Trigger for Robertsonian Translocations and Speciation
Source: Int J Mol Sci. 2020 Nov 23;21(22):8871. doi: 10.3390/ijms21228871 (PMC7700478; doi:10.3390/ijms21228871)
Supplement: Supplementary file 1 [file ijms-21-08871-s001.zip › Figure_S2.pdf]

A

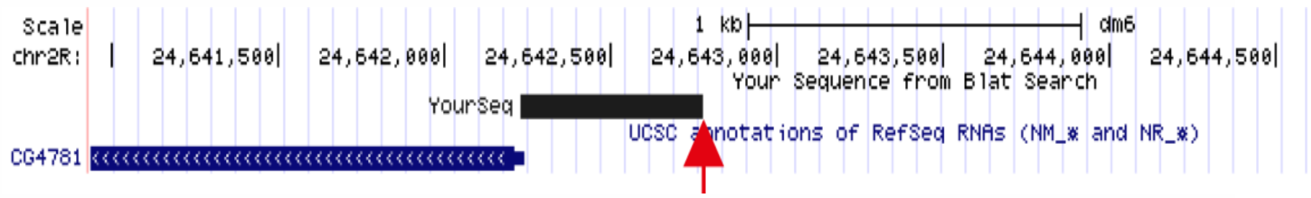

B

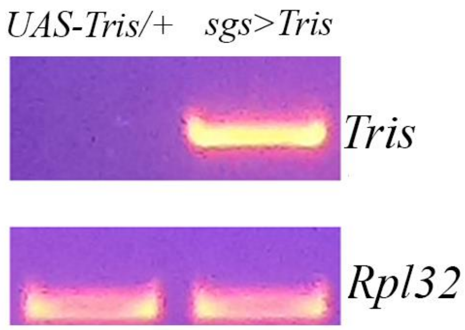

Figure S2. Mapping of *UAS-Tris* transgenic construct. (**a**) Using inverse PCR we found only one copy of *UAS-Tris* transgenic construct that was located 539 bp upstream of *CG4781* 5'-termini on chromosome 2R. The red arrow indicates the position of *UAS-Tris* insertion in the map. The expression levels of *CG4781* were not changed upon insertion and overexpression of *Trf2*-knockdown transgene as evaluated by RT-PCR in preliminary experiments. (**b**) *UAS*-mediated inducible overexpression of *Trf2* knock-down transgene was confirmed using RT-PCR with primer for *hsp70* promoter located in *pUAST* vector and one of primers used for *Trf2* knock-down transgene construction (Trf2XbaI). Total RNA for evaluating of transgene expression was extracted from salivary glands of *UAS-Tris*/+ (control) and *sgs-Gal4>Uas-Tris* third instar larvae. *Rpl32* gene was used as the endogenous control.
