# Supplementary material for: TBP-Related Factor 2 as a Trigger for Robertsonian Translocations and Speciation
Source: Int J Mol Sci. 2020 Nov 23;21(22):8871. doi: 10.3390/ijms21228871 (PMC7700478; doi:10.3390/ijms21228871)
Supplement: Supplementary file 1 [file ijms-21-08871-s001.zip › Table_S1.pdf]

Table 1. X-chromosome nondisjunction and existence of attached-X chromosomes in females with lethal *Trf2* mutations.

First column indicates ordinal number of parent females. Next two columns indicate the amount of regular F1 progeny from *l(1)/FM/Y(?) x y/Y* crosses: X/X, total amount of females with $y^+l(1)B^+/yB^+$ and yB/yB^+ genotypes; X/Yx2, doubled amount of males with *yB/Y* genotype. Next two columns indicate the amount of exceptional F1 progeny: X/0, males with $yB^+/0$ genotype; XX/Y or X^X/Y, females with $y^+l(1)B^+/yB/Y$ genotype. Q_n - Frequency of X-chromosome nondisjunction, calculated as described in Materials and Methods.

Ordinal number of parent females	Regular progeny		Exceptional progeny	
	♀♀ X/X	♂♂ X/Yx2	♀♀ XX/Y (or X^X/Y)	♂♂ X/0
***l(1)G0425;* Q_n=7.18%**				
**1.**	83	82	0	0
**2.**	51	44	1	1
**3.**	**0**	**0**	**30**	**30**
**4.**	**0**	**0**	**16**	**27**
**5.**	52	52		
**6.**	**0**	**0**	**30**	**35**
**7.**	51	18	0	0
**8.**	42	54	0	0
**9.**	**0**	**0**	**19**	**15**
**10.**	**0**	**0**	**20**	**20**
**11.**	45	40	0	0
**12.**	32	26	1	1
**13.**	66	50	2	3
**14.**	30	26	0	2
**15.**	10	3	0	0
**16.**	43	11	0	0
**17.**	36	22	1	1
**18.**	**0**	**0**	**40**	**23**
**19.**	95	26	0	0
**20.**	14	3	0	0
**21.**	80	70	1	4
**22.**	30	24	1	1
**23.**	39	28	1	1
**24.**	25	22	1	3
**25.**	27	22	1	2
**Total***	**416**	**334**	**10**	**19**
***l(1)G0356;* Q_n=11.59%**				
**1.**	50	30	0	0
**2.**	99	64	0	0
**3.**	44	6	0	0
**4.**	100	76	1	4
**5.**	71	64	0	0

**6.**	100	90	5	7
**7.**	70	56	0	0
**8.**	**0**	**0**	**40**	**35**
**9.**	50	54	0	0
**10.**	65	74	0	0
**11.**	**0**	**0**	**29**	**31**
**12.**	38	46	0	0
**13.**	40	12	0	0
**14.**	28	16	1	2
**15.**	31	20	0	3
**16.**	21	24	3	2
**17.**	26	22	1	1
**18.**	14	14	3	3
**19.**	23	26	1	1
**20.**	41	20	1	2
**21.**	57	72	0	0
**22.**	73	52	0	0
**23.**	39	28	0	0
**24.**	20	12	1	2
**25.**	18	16	1	2
**26.**	23	12	3	2
**Total***	**445**	**348**	**21**	**31**
***l(1)G0424;* Q_n=17.39%**				
**1.**	54	44	6	4
**2.**	55	56	8	5
**3.**	64	54	0	0
**4.**	55	60	0	0
**5.**	37	60	5	1
**6.**	53	58	0	0
**7.**	53	38	0	0
**8.**	46	66	0	0
**9.**	69	66	0	0
**10.**	46	46	3	2
**11.**	22	30	2	2
**12.**	32	38	0	0
**13.**	38	34	0	0
**14.**	23	16	3	2
**15.**	18	20	0	0
**16.**	22	28	7	4
**17.**	17	24	1	3
**18.**	20	18	0	0
**19.**	42	50	0	0
**20.**	25	26	4	3
**21.**	24	16	0	0
**22.**	41	38	0	0
**23.**	36	36	0	0
**24.**	36	34	0	0
**25.**	42	30	4	5
**Total***	**343**	**360**	**43**	**31**

l(1)G0295; **Q_n=14,51%**				
**1.**	22	20	0	0
**2.**	40	24	4	2
**3.**	16	22	0	0
**4.**	12	10	0	0
**5.**	28	36	0	0
**6.**	24	22	3	3
**7.**	26	32	0	0
**8.**	31	26	2	3
**9.**	35	42	3	3
**10.**	36	38	0	0
**11.**	27	26	0	0
**12.**	24	16	4	3
**13.**	54	42	0	0
**14.**	58	44	0	0
**15.**	47	52	0	0
**16.**	50	60	6	3
**17.**	56	34	5	3
**18.**	67	76	0	0
**19.**	65	46	4	3
**20.**	59	46	0	0
**21.**	64	64	7	6
**22.**	59	62	0	0
**23.**	43	58	2	2
**24.**	45	42	0	0
**25.**	46	48	3	4
**26.**	55	36	6	5
**27.**	44	54	3	2
**Total***	**577**	**530**	**52**	**42**
l(1)G0166; **Q_n=29.6%**				
**1.**	8	12	0	0
**2.**	17	10	0	0
**3.**	15	21	0	0
**4.**	21	16	5	2
**5.**	23	18	0	0
**6.**	20	14	0	0
**7.**	18	14	0	0
**8.**	19	20	3	3
**9.**	16	12	0	0
**10.**	22	26	0	0
**11.**	18	18	0	0
**12.**	17	24	0	0
**13.**	13	20	0	0
**14.**	21	16	8	4
**15.**	17	8	0	0
**16.**	18	12	4	2
**17.**	24	24	0	0
**18.**	19	22	0	0
**19.**	20	14	0	0

**20.**	12	10	1	2
**21.**	12	18	0	0
**22.**	11	28	0	0
**23.**	21	14	0	0
**24.**	17	22	0	0
**25.**	17	10	0	0
26.	17	6	0	0
27.	14	16	4	3
28.	10	22	0	0
29.	18	16	0	0
30.	11	6	0	0
**Total***	**105**	**90**	**25**	**16**
***l(1)G0152;* Q$_n$=28.25%**				
**1.**	10	10	4	3
**2.**	18	20	4	5
**3.**	23	22	0	0
**4.**	24	14	0	0
**5.**	22	12	0	0
**6.**	12	26	3	2
**7.**	7	20	0	0
**8.**	19	18	0	0
**9.**	21	8	0	0
**10.**	16	10	1	2
**11.**	25	24	0	0
**12.**	8	16	0	0
**13.**	13	18	0	0
**14.**	17	12	4	4
**15.**	17	22	2	3
**16.**	21	16	0	0
**17.**	14	28	0	0
**18.**	21	14	0	0
**19.**	16	20	3	5
**20.**	11	8	0	0
**21.**	22	6	0	0
**22.**	22	20	0	0
**23.**	15	26	0	0
**24.**	11	22	2	4
**25.**	26	20	0	0
**26.**	15	12	0	0
**27.**	6	0	0	0
**28.**	21	16	0	0
**29.**	13	24	0	0
**30.**	27	16	0	0
**Total***	**117**	**142**	**23**	**28**
***l(1)EF520;* Q$_n$=29.12%**				
**1.**	15	20	0	0
**2.**	17	16	0	0
**3.**	13	12	0	0

**4.**	22	16	0	0
**5.**	12	10	0	0
**6.**	20	20	0	0
**7.**	17	8	0	0
**8.**	25	16	3	4
**9.**	18	18	0	0
**10.**	22	26	0	0
**11.**	11	28	0	0
**12.**	11	14	0	0
**13.**	18	10	0	0
**14.**	12	20	0	0
**15.**	15	6	0	0
**16.**	18	14	5	3
**17.**	27	22	0	0
**18.**	20	20	0	0
**19.**	24	18	0	0
**20.**	15	26	0	0
**21.**	15	16	0	0
**22.**	16	22	0	0
**23.**	10	14	0	0
**24.**	11	8	0	0
**25.**	13	16	0	0
**26.**	28	12	0	0
**27.**	16	24	0	0
**28.**	24	20	0	0
**29.**	13	18	0	0
**30.**	22	24	0	0
**Total***	**43**	**30**	**8**	**7**
Control lines				
In(1)FM Q_n=**1.4%**	1242	1186	6	11
l(1)G0071 Q_n=**1.5%**	363	282	1	4

Note: Data obtained from parental females with attached-X chromosomes are marked in red. Rows with data obtained from parental females with normal chromosome segregation are marked with a gray background. * - The total number of progeny obtained from only those females who produced regular and exceptional offspring simultaneously.
